# Haploid induction in sweet potato by activating the AP2/ERF family transcription factor *IbBBM*

**DOI:** 10.1111/pbi.70137

**Published:** 2025-05-14

**Authors:** Wenpeng Song, Yixuan Zhu, Junyao Zheng, Hong Zhai, Shaozhen He, Huan Zhang, Ning Zhao, Qingchang Liu, Shaopei Gao

**Affiliations:** ^1^ Key Laboratory of Sweet Potato Biology and Biotechnology of Ministry of Agriculture and Rural Affairs College of Agronomy & Biotechnology, China Agricultural University Beijing China

**Keywords:** sweet potato, AP2/ERF transcription factor, CRISPRa, *IbBBM*, haploid

Sweet potato (*Ipomoea batatas* L.) is a major root crop in the world. As an asexual crop, sweet potato breeding and basic research face a number of challenges due to the highly heterozygous genome. Doubled haploid (DH) breeding is one of the most efficient means of improving crop germplasm. However, there is no reliable haploid induction system for sweet potato. *BABY BOOM* (*BBM*) encodes an AINTEGUMENTA‐LIKE (AIL) APETALA2/ethylene‐responsive factor (AP2/ERF) domain transcription factor (Boutilier *et al*., 2002). It is a key regulator of plant cell totipotency and induces asexual embryo formation when ectopically expressed (Chen *et al*., 2022). This property has been widely exploited to improve plant transformation and clonal propagation. Nevertheless, the ability of the *BBM* homologue to induce parthenogenesis and produce viable haploid plants in sweet potato remains to be investigated.

To identify a candidate for a sweet potato BBM homologue, a tblastn analysis was performed against the sweet potato genome (Wu *et al*., 2024; Yang *et al*., 2017; Yoon *et al*., 2022) using the Arabidopsis BBM protein sequence (AT5G17430) as a query. The most likely candidates were then used for a phylogenetic study. Phylogenetic analysis revealed that candidate Ibat.Tzn_v2.02EG002520.1 (termed IbBBM) is the closest homologue of AtBBM and BnBBMs (Figures 1a and S1). Due to the highly heterogeneous nature of the sweet potato genome (2*n* = B_1_B_1_B_2_B_2_B_2_B_2_ = 6*x* = 90), sequence variations among alleles of the same gene are common, making it difficult to choose which allele to clone and overexpress. CRISPR activation (CRISPRa), based on deactivated Cas (dCas) proteins coupled to activator domains, offers a promising alternative to the conventional approach of gene overexpression (Konermann *et al*., 2015). Although CRISPRa technologies are now available in plants, the applications of transcriptional activation in dicotyledonous plants, especially sweet potato, are still scarce. Firstly, we developed a CRISPR‐dCas9‐6×TAL‐2×VP64 system capable of inducing target gene activation in sweet potato (Figure 1b). Next, we designed a single‐guide RNA (sgRNA) specific for a conserved region upstream of the *IbBBM* start codon (ATG) and introduced the construct into the sweet potato variety Xushu27 via *Agrobacterium*‐mediated transformation (Figure 1c; Figure S2). Three *IbBBM‐*CRISPRa lines were transgenic positive and showed high levels of *IbBBM* expression (Figure 1d,e). Compared to the wild type (WT), the transgenic positive lines did not show any obvious changes in flowering time, vine length, and stem base thickness, but they produced more seeds (Figure 1f,g; Figure S3). A total of 49 seeds were harvested from the *IbBBM*‐CRISPRa transgenic‐positive lines, whereas only 13 were harvested from the WT and the negative lines (Figure 1g; Table S1). Sweet potato is self‐incompatible, so more seeds produced in transgenic plants may be the result of induced parthenogenesis following *IbBBM* activation. Furthermore, the harvested seeds were germinated to assess the presence of haploids in the progeny. All seed‐germinated plants except two (#4–7 and #6–10) showed typical hexaploid plant phenotypes. Compared to the hexaploid control (Mock), the main stems of plants #4–7 and #6–10 became slender and weak (Figure 1h). Previous studies have shown that genome size is positively related to guard cell size (Beaulieu *et al*., 2008). Microscopic observations of leaf epidermal cells revealed that the guard cell size was reduced in #4–7 and #6–10 (19.4 ± 1.3 μm) compared to Mock (27.6 ± 0.8 μm) (Figure 1i; Figure S4). Flow cytometry was then performed on all seed‐germinated plants to confirm their ploidy level. As expected, the signal intensity values in #4–7 and #6–10 were approximately half those of the Mock (Figure 1j; Figure S5a). Furthermore, it was further confirmed that both #4–7 and #6–10 were haploid using the known hexaploid sweet potato variety Xushu 18 as a reference (Yoon *et al*., 2022) (Figure S5b,c). By contrast, no haploid plants were found among the germinated seeds produced by WT and the negative lines. Here, we propose a model for the induction of sweet potato haploid formation through the activation of *IbBBM* (Figure 1k).

**Figure 1:**
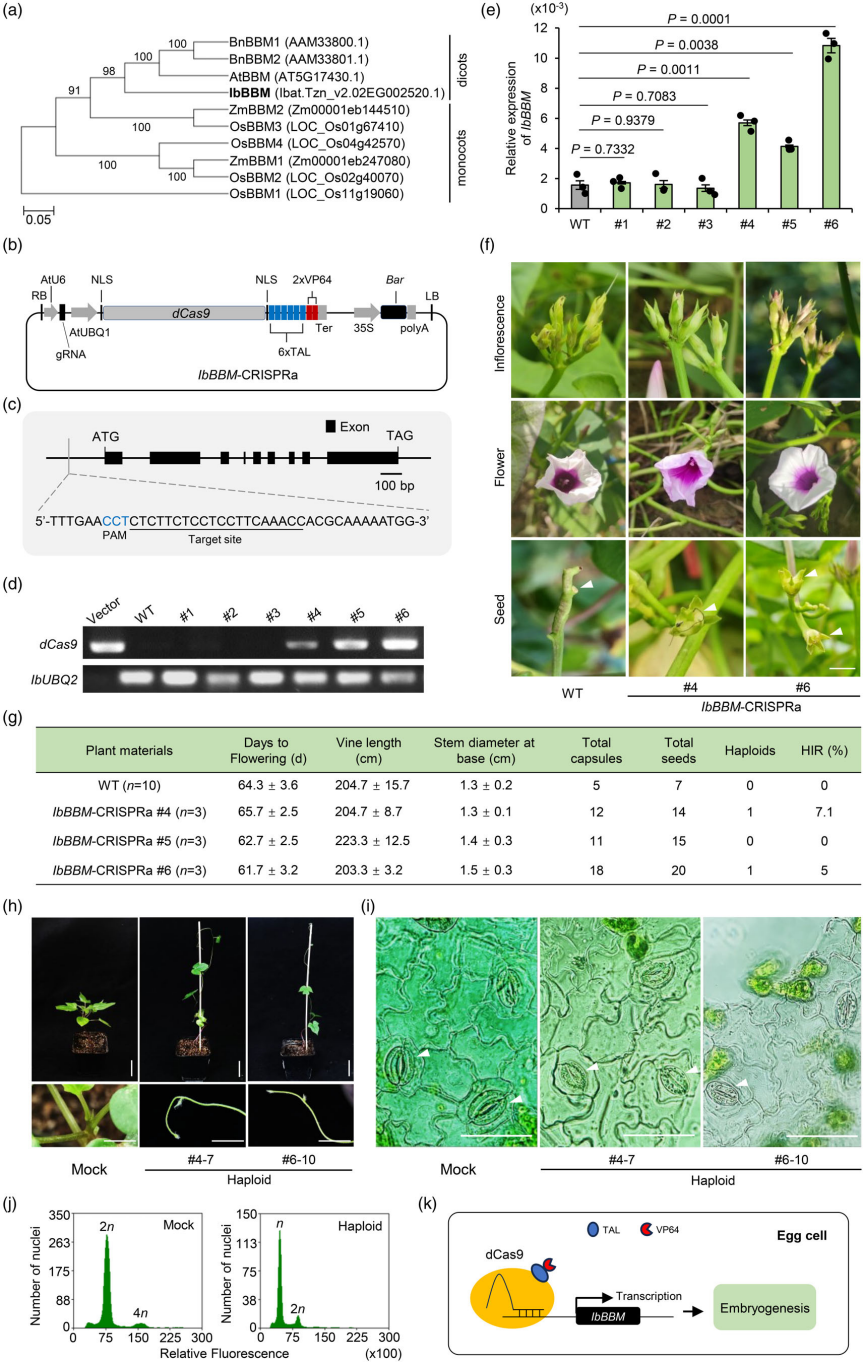
Haploid induction caused by the targeted activation of *IbBBM* in vivo. (a) Phylogenetic tree of BBM proteins from various species, including *Arabidopsis thaliana* (At), *Brassica napus* (Bn), *Zea mays* (Zm), *Oryza sativa* (Os) and *Ipomoea batatas* (Ib). The phylogenetic tree was constructed using the neighbour‐joining method with MEGA11 software. Numbers next to the branches indicate bootstrap value percentages from 1000 replications. (b) Constructed vector of *IbBBM*‐CRISPRa. NLS, nuclear localization signal; Ter, terminator; LB, left border; RB, right border; polyA, CaMV poly (A) terminator sequence. TAL, TAL effector transcription activation domain. (c) The genomic structure of *IbBBM* and the gRNA for the CRISPRa system. PAM, protospacer adjacent motif. The PAM site is marked in blue font and the target site is underlined. (d) Identification of *IbBBM*‐CRISPRa sweet potato transgenic plants. Vector, *IbBBM*‐CRISPRa plasmid; WT, wild type. (e) Relative mRNA levels of *IbBBM* were quantified by reverse transcription quantitative PCR in leaves. The sweet potato ubiquitin gene *IbUBQ2* was used as an internal reference. The data are presented as the mean ± SD (*n* = 3). (f) Inflorescences, flowers and seeds of WT and *IbBBM*‐CRISPRa lines. *n*, the number of plants used for statistical agronomic traits. Scale bars: 2 cm. (g) Analysis of the number of days to flowering, vine length, stem diameter at the base, and the total number of capsules, seeds and haploids in WT and *IbBBM*‐CRISPRa plants. HIR, haploid induction rate. (h) Morphology and stem tip of the hexaploid control (Mock) and the haploids. Scale bars: 5 cm. (i) Pictorial representation of stomata size in the lower epidermis of the Xushu 27 (Mock) and haploids. Scale bars: 60 μm. (j) Ploidy levels of the haploid (#4–7) and the Xushu 27 (Mock) determined by flow cytometry. (k) Proposed model for the dCas9‐mediated activation of *IbBBM* in the sweet potato egg cells to induce haploid formation. Nuclease‐deficient Cas9 (dCas9) is fused to a transcriptional activator and targeted to the *IbBBM* promoter. Activated expression of *IbBBM* in the sweet potato egg cell promotes embryogenesis, induction of parthenogenesis and subsequent production of haploids.

As an asexual crop, hybridization and selection remain the primary methods of sweet potato breeding. However, the varieties currently used in sweet potato production practices are mainly derived from inter‐varietal hybrids. With the development and innovation of plant haploid induction technologies, haploid breeding will be widely used in the varietal improvement of sweet potato. Sweet potatoes can naturally flower and set seed south of latitude 23° N latitude but generally do not flower in northern China. In this study, we selected a variety, Xushu27, which flowers naturally in Beijing, and used constitutive activation of *IbBBM*. More locations, varieties, and tissue‐specific activation methods will be tested in future studies. Altogether, these results provide a favourable reference for inducing efficient parthenogenesis and forming viable haploid plants in sweet potato.

## Conflict of interest

The authors have declared no conflict of interest.

## Author contributions

S.G. conceived the idea and designed the experiments. W.S. performed most of the experiments and analyses. S.G. constructed the vector with contributions from J.Z. and Y.Z. Y.Z. created the transgenic materials and contributed to the field experiments. H.Z., H.Z., N.Z., S.H. and Q.L. contributed to the acquisition of funding and supervised the project. W.S. drafted the original manuscript. S.G. revised and finalized the manuscript. All authors read and approved the final version of the manuscript.

## Supporting information


**Figure S1** Multiple protein sequence alignment of AtBBM, IbBBM and IbPLT, with conserved amino acids shaded in different colours.
**Figure S2** Sanger sequencing chromatogram of the CRISPRa target site of the *IbBBM* promoter in wild‐type Xushu27.
**Figure S3** Inflorescences, flowers and seeds of negative lines.
**Figure S4** Bar graph showing the length of stomatal guard cells in Mock and haploid plants.
**Figure S5** Flow‐cytometric DNA histograms for ploidy determination.
**Table S1** Analysis of the number of days to flowering, vine length, stem diameter at the base and the total number of capsules, seeds and haploids in the negative lines.
**Table S2** Primers used in this study.

## Data Availability

All data in this study are available in this article or supplementary information.
