# Supplementary material for: Haploid induction in sweet potato by activating the AP2/ERF family transcription factor IbBBM
Source: Plant Biotechnol J. 2025 May 14;23(8):3113–5. doi: 10.1111/pbi.70137 (PMC12310834; doi:10.1111/pbi.70137)
Supplement: Supplementary file 1 — Figure S1 Multiple protein sequence alignment of AtBBM, IbBBM and IbPLT, with conserved amino acids shaded in different colours. Figure S2 Sanger sequencing chromatogram of the CRISPRa target site of the IbBBM promoter in wild‐type Xushu27. Figure S3 Inflorescences, flowers and seeds of negative lines. Figure S4 Bar graph showing the length of stomatal guard cells in Mock and haploid plants. Figure S5 Flow‐cytometric DNA histograms for ploidy determination. Table S1 Analysis of the number of days to flowering, vine length, stem diameter at the base and the total number of capsules, seeds and haploids in the negative lines. Table S2 Primers used in this study. [file PBI-23-3113-s001.docx]

**Supplemental Materials**

**Materials and Methods**

**Plant materials and growth conditions**

The sweet potato variety Xushu27 was used as the wild type in this study. Wild-type and transgenic sweet potato plants were grown in the greenhouse or in the experimental base of China Agricultural University. The greenhouse growth conditions included natural light supplemented by LED lighting (12 h: 12 h photoperiod), a humidity level of 60%, and temperatures of 28°C during the day and 20°C at night. For the field experiment, sweet potato vine cuttings were transplanted in the field at a 25×90 cm spacing using a ridge planting method. The ridge height was about 30 cm, and the ridge width was about 40 cm. Normal field management, including irrigation, fertilization and disease control, was carried out according to normal sweet potato cultivation practices.

**Vector construction** **for plant transformation**

To obtain the CRISPRa vector, the sequences of the Arabidopsis *U6-26* promoter (*AtU6*) and gRNA scaffold, the Arabidopsis ubiquitin (*AtUBQ*, AT3G52590) promoter, the nuclear localization signal (NLS), dCas9 (D10A, H830A), a transcriptional activation domain (6×TAL-2×VP64) and the E9 terminator were amplified or synthesized (Tsingke, China) and assembled into pCAMBIA3300 (CAMBIA, Canberra, Australia).

To construct *IbBBM*-CRISPRa expression vectors, single-stranded DNA oligos of gRNA sequences (gIbBBM-F and gIbBBM-R) were synthesized. Then they were annealed and cloned into the downstream of an *AtU6* promoter using *Bsa* I enzyme. The construct was confirmed by Sanger sequencing. The primers used in this study are listed in Supplemental Table 2.

***Agrobacterium* *tumefaciens*-mediated in planta transformation**

A positive single colony of *Agrobacterium tumefaciens* strain EHA105 was picked from the plate and transferred to 500 μL of liquid LB (containing 50 mg/L kanamycin and 20 mg/L rifampin). The mixture was subsequently incubated at 250 rpm for 12 h at 28°C to obtain the starter culture. Next, 100 μL of the starter culture was transferred to 100 mL of LB medium and incubated at 28°C with shaking at 250 rpm until an optical density at 600 nm (OD_600_) of 1.2 was reached.

Wild-type Xushu27 were used as the recipients for *Agrobacterium*-mediated transformation as described previously (Zhang et al., 2023). Briefly, the adventitious roots of sweet potato vine cuttings were removed prior to inoculation with the *Agrobacterium tumefaciens*. The seedlings were infected with *Agrobacterium tumefaciens* EHA105 by wounding the nodes with a syringe. The top 25-30 cm of the sweet potato vines were selected, the leaves and petioles of the basal 3-4 nodes and any adventitious roots were removed and the nodes were pierced. The treated sweet potato stem nodes were immersed in the *Agrobacterium tumefaciens* solution and left to infect for 8-12 h. During this time, gentle shaking of the solution ensured better infestation of the stems. The infected stem segments were then placed in triangular flasks filled with distilled water in a morphological orientation for 1 day in the dark. The stem segments were then subjected to bacteriostatic inhibition with the cefotaxime at a concentration of 200 mg/L in the working solution. The stem segments were rinsed twice with distilled water and transplanted to the field.

**Identification of positive transgenic plants**

Genomic DNA from sweet potato leaves was extracted using the cetyltrimethylammonium bromide (CTAB) method. Specific primers (dCas9-F and dCas9-R) were designed to detect the presence of the dCas9 gene. At the same time, specific primers (IbUBQ2-F and IbUBQ2-R) for sweet potato ubiquitin were designed as an internal reference. The primers are listed in Supplementary Table S2.

**Quantitative PCR and data analysis**

Total RNA was extracted from leaves using TRIzon reagent (Cwbio, Beijing, China) according to the manufacturer’s instructions. One microgram of total RNA was treated with DNase I and used for cDNA synthesis using the HiFiScript gDNA Removal cDNA Synthesis Kit (CwBio, Beijing, China). Gene expression was analyzed using the StepOnePlus^TM^ Real-Time PCR system (Applied Biosystems) according to the manufacturer’s instructions. PCR cycle conditions were 95°C for 5 min as the first denaturing step, followed by 40 cycles at 95°C for 10 s, 60°C for 30 s, and a gradual increase in temperature from 60 to 95°C during the dissociation stage to monitor the specificity of each primer pair. The sweet potato ubiquitin gene *IbUBQ2* was used as an internal reference. Three biological replicates were performed for each analysis. Relative expression levels were calculated using the 2^-ΔΔCt^ method (Schmittgen et al., 2008). Data are normalized to *IbUBQ2* mRNA expression. The primers used for quantitative PCR are listed in Supplementary Table S2.

**Stomatal measurement**

Leaf epidermis from 6-week-old haploid and hexaploid controls (Mock) was obtained by peeling and prepared into slides (approximately 2×2 cm in size). Images were captured using a fluorescence microscope (Revolve; Echo, San Diego, CA, USA).

**Flow cytometry analysis**

Samples were analysed using a CyFlow Ploidy Analyser flow cytometer (Sysmex-Partec GmbH, Gorlitz, Germany). Samples were disrupted with a razor blade for 40 seconds in 250 μL CyStain UV Precise P Nuclei Extraction Buffer. Nuclei were passed through a 50 μM filter and mixed with 1 mL of CyStain UV Precise P staining buffer. The gain was set at 540, the speed at 4 μL/s, and the sample concentration was established to be ~1,500 cells per millilitre. Data were acquired and analyzed using the FCS express software. With the first signal peak at ~7,500 (Relative Fluorescence value), was used as a diploid control. Samples with the first signal peak at ~3,750 (relative fluorescence value) were deemed to be haploids. The detailed operation steps following the protocol described by Kelliher et al. in 2017.

**Accession numbers**

The sweet potato sequence of *IbBBM* (Ibat.Tzn_v2.02EG002520.1), *IbPLT* (Ibat.Tzn_v2.14AG007090.1) and *IbUBQ2* (Ibat.Tzn_v2.05AG027230.2) can be found in the *I. batatas* ‘Tanzania’ genome database (Wu et al., 2024). The genome database websites for other species are as follows: Arabidopsis (https://www.arabidopsis.org/); Maize (https://maizegdb.org/); Rice (<https://riceome.hzau.edu.cn/>).

**References**

Kelliher T., Starr D., Richbourg L., Chintamanani S., Delzer B., Nuccio M.L., Green J. et al. (2017) MATRILINEAL, a sperm-specific phospholipase, triggers maize haploid induction. *Nature*, 542, 105-109.

Schmittgen T.D. and Livak K.J. (2008) Analyzing real-time PCR data by the comparative C_T_ method. *Nat. Protoc.* 3, 1101-1108.

Wu, S., Sun, H., Hamilton, J.P., Mollinari, M., Gesteira, G.D.S., Kitavi, M., Yan M. et al. (2024) Phased chromosome-level genome assembly provides insight into the origin of hexaploid sweetpotato. *bioRxiv*, doi:10.1101/2024.08.17.608395.

Zhang W., Zuo Z., Zhu Y., Feng Y., Wang Y., Zhao H., Zhao N. et al. (2023) Fast track to obtain heritable transgenic sweet potato inspired by its evolutionary history as a naturally transgenic plant. *Plant Biotechnol. J*. 21, 671-673.

**Supplemental figures**

**
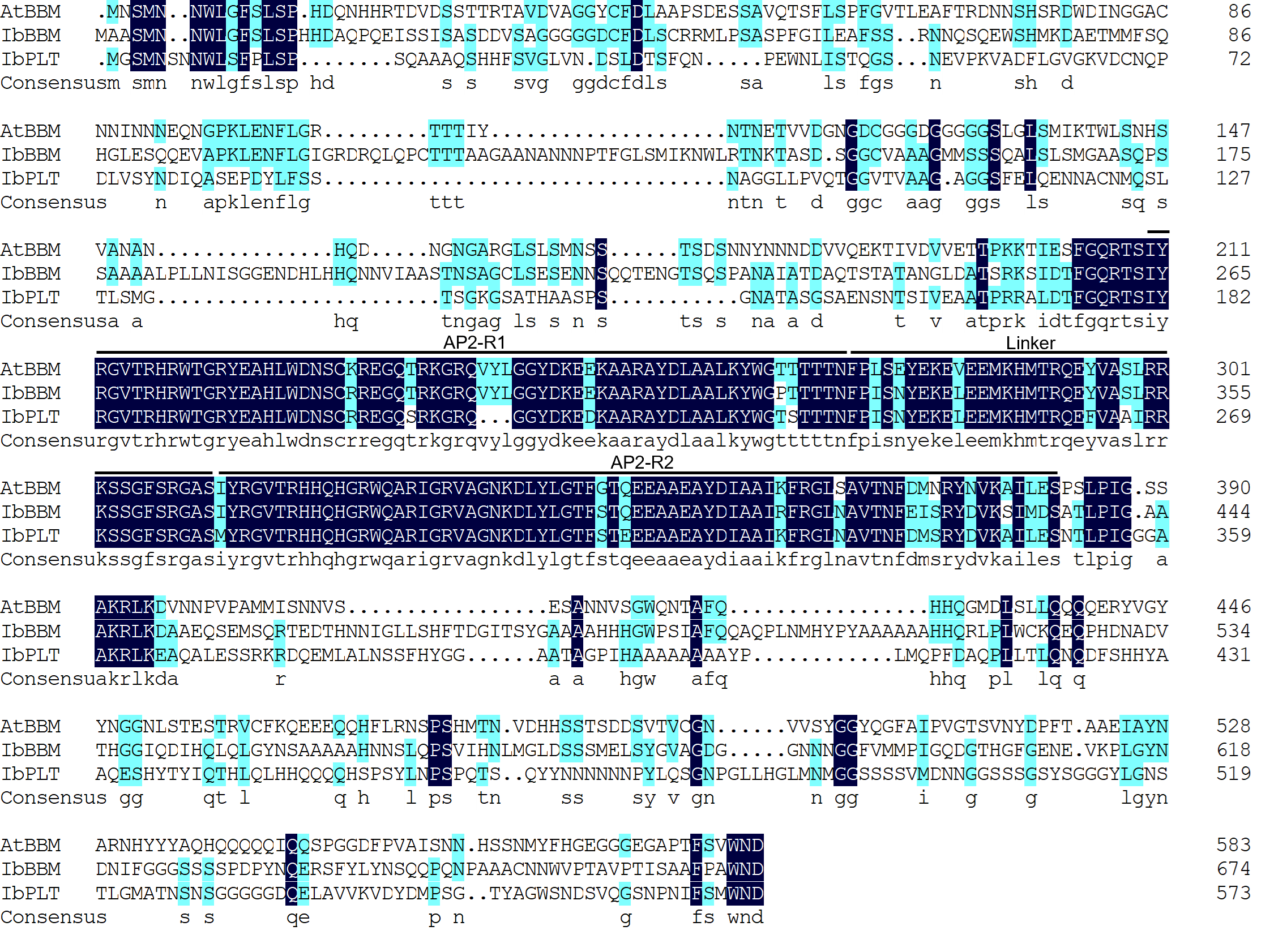
**

**Figure S1** Multiple protein sequence alignment of AtBBM, IbBBM and IbPLT, with conserved amino acids shaded in different colors. The entire lines represented the conserved AP2 domains (AP2-R1 and AP2-R2), which are separated by a linker.


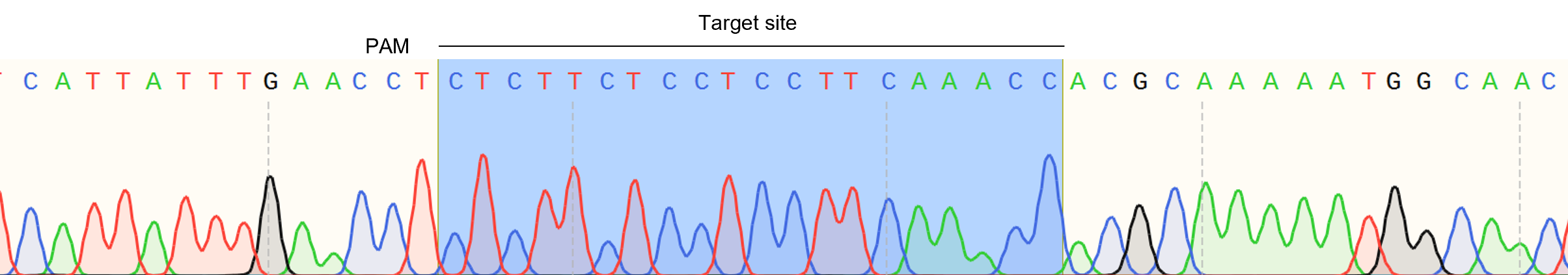


**Fig****ure S2** Sanger sequencing chromatogram of the CRISPRa target site of the *IbBBM* promoter in wild-type Xushu27. SgIbBBM-F and SgIbBBM-R are the forward and reverse primers used for the PCR, and the SgIbBBM-F primer is used for Sanger sequencing.


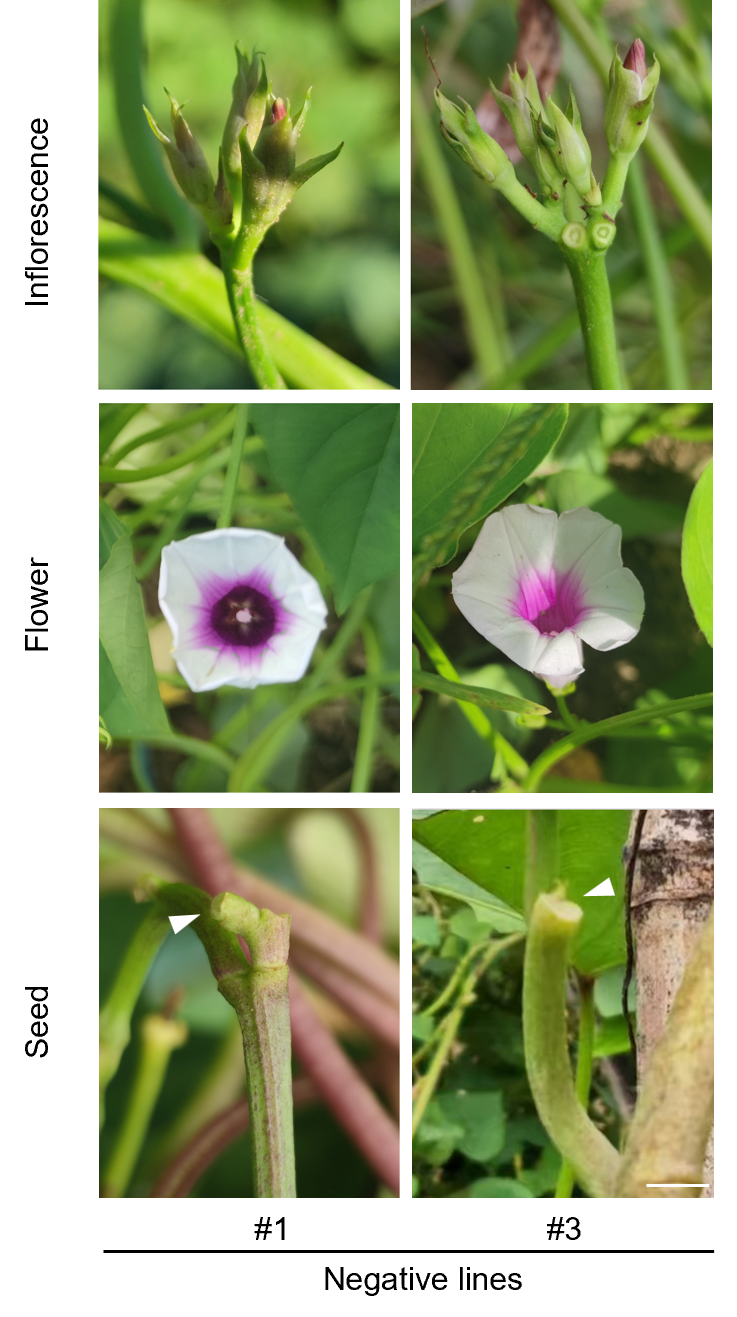


**Figure S3** Inflorescences, flowers and seeds of negative lines. Scale bars: 2 cm.


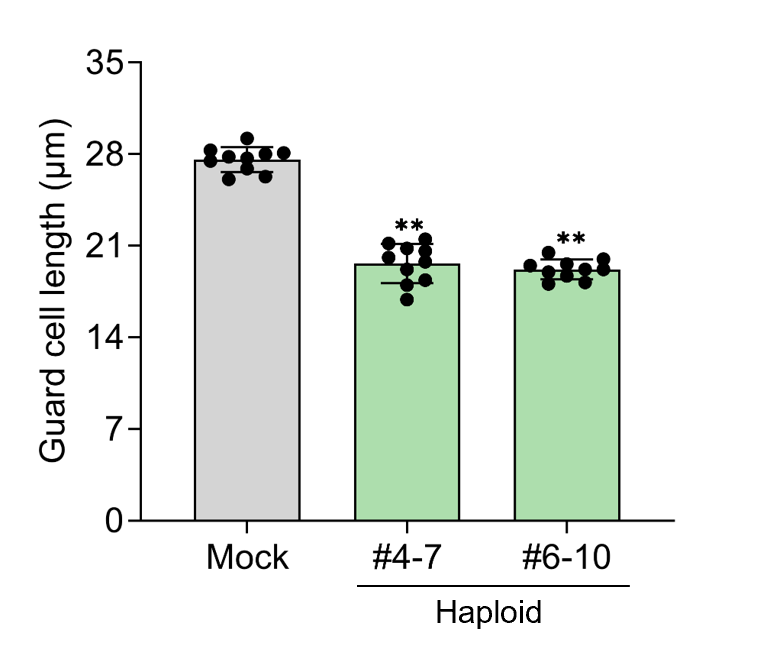


**Figure S4** Bar graph showing the length of stomatal guard cells in Mock and haploid plants. All data are presented as means ± SD (*n* = 3). **, *P* < 0.01; Student’s *t*-test.


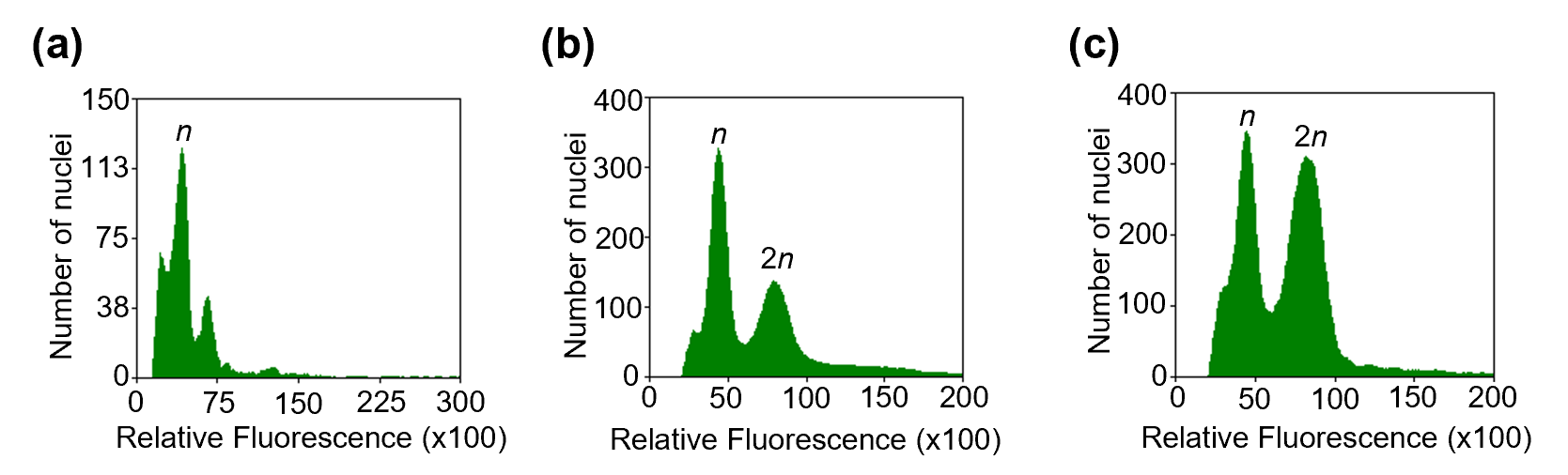


**Figure** **S5** Flow-cytometric DNA histograms for ploidy determination. Parthenogenetic haploid (#6-10) showing a 1*n p*eak (a), the mixed samples of #4-7 and hexaploid showing 1*n* and 2*n* peaks (b) and the mixed samples of #6-10 and hexaploid showing 1*n* and 2*n* peaks (c). The hexaploid used in (b) and (c) is hexaploid sweet potato variety Xushu 18 (2n=6x=90).

**Supplemental tables**

**Table S1** Analysis of the number of days to flowering, vine length, stem diameter at the base, and the total number of capsules, seeds, and haploids in the negative lines.

| Plant materials | Days to  Flowering (d) | Vine length  (cm) | Stem diameter at base (cm) | Total  capsules | Total  seeds | Haploids | HIR (%) |
| --- | --- | --- | --- | --- | --- | --- | --- |
| #1 (*n*=3) | 64.7 ± 3.1 | 200.7 ± 18.6 | 1.6 ± 0.4 | 2 | 2 | 0 | 0 |
| #2 (*n*=3) | 62.3 ± 5.1 | 212.7 ± 10.1 | 1.4 ± 0.2 | 1 | 2 | 0 | 0 |
| #3 (*n*=3) | 68.3 ± 3.5 | 207.3 ± 9.5 | 1.4 ± 0.4 | 1 | 2 | 0 | 0 |

*n*, the number of plants used for statistical agronomic traits.

**Table S2** Primers used in this study.

| Primer name | Sequence |
| --- | --- |
| SgIbBBM-F | 5'-ACTTTGCCTTGGAAGAAATGAC-3' |
| SgIbBBM-R | 5'-GCATGGAAAGACGGTGAAGT-3' |
| gIbBBM-F | 5'-GATTGGTTTGAAGGAGGAGAAGAG-3' |
| gIbBBM-R | 5'-AAACCTCTTCTCCTCCTTCAAACC-3' |
| dCas9-F | 5'-ATCCACGACGACTCCCTCAC-3' |
| dCas9-R | 5'-CCTTCTGGGTGGTTTGGTTC-3' |
| IbUBQ2-F | 5'-CACCTTGTCCTTCGTCTCCGT-3' |
| IbUBQ2-R | 5'-AGGGAACATCAAACAGCAGATAACAG-3' |
| qIbBBM-F | 5'-ATGATGCCGATTGGACAGGA-3' |
| qIbBBM-R | 5'-GGTTGCTGGGAGTTGTAGAGGT-3' |
| qIbUBQ2-F | 5'-CATCCAGAAGGAGTCTACCCTG-3' |
| qIbUBQ2-R | 5'-CAGAAGGGAACATCAAACAGCAGAT-3' |
